# Supplementary material for: Galectin-1 and platelet factor 4 (CXCL4) induce complementary platelet responses in vitro
Source: PLoS One. 2021 Jan 7;16(1):e0244736. doi: 10.1371/journal.pone.0244736 (PMC7790394; doi:10.1371/journal.pone.0244736)
Supplement: S1 Fig — Bar graphs represent the percentage of platelet αIIbβ3 activation (A), P-selectin expression (B) and PS-exposure (C) by gal-1 in the absence or presence of heparin (10μg/mL). Mean ± SD (n = 4-12). *p < 0.05, **p < 0.01, ***p < 0.001 as compared to control (no gal-1, Kruskal Wallis/Dunn’s test). (DOCX) [file pone.0244736.s001.docx]

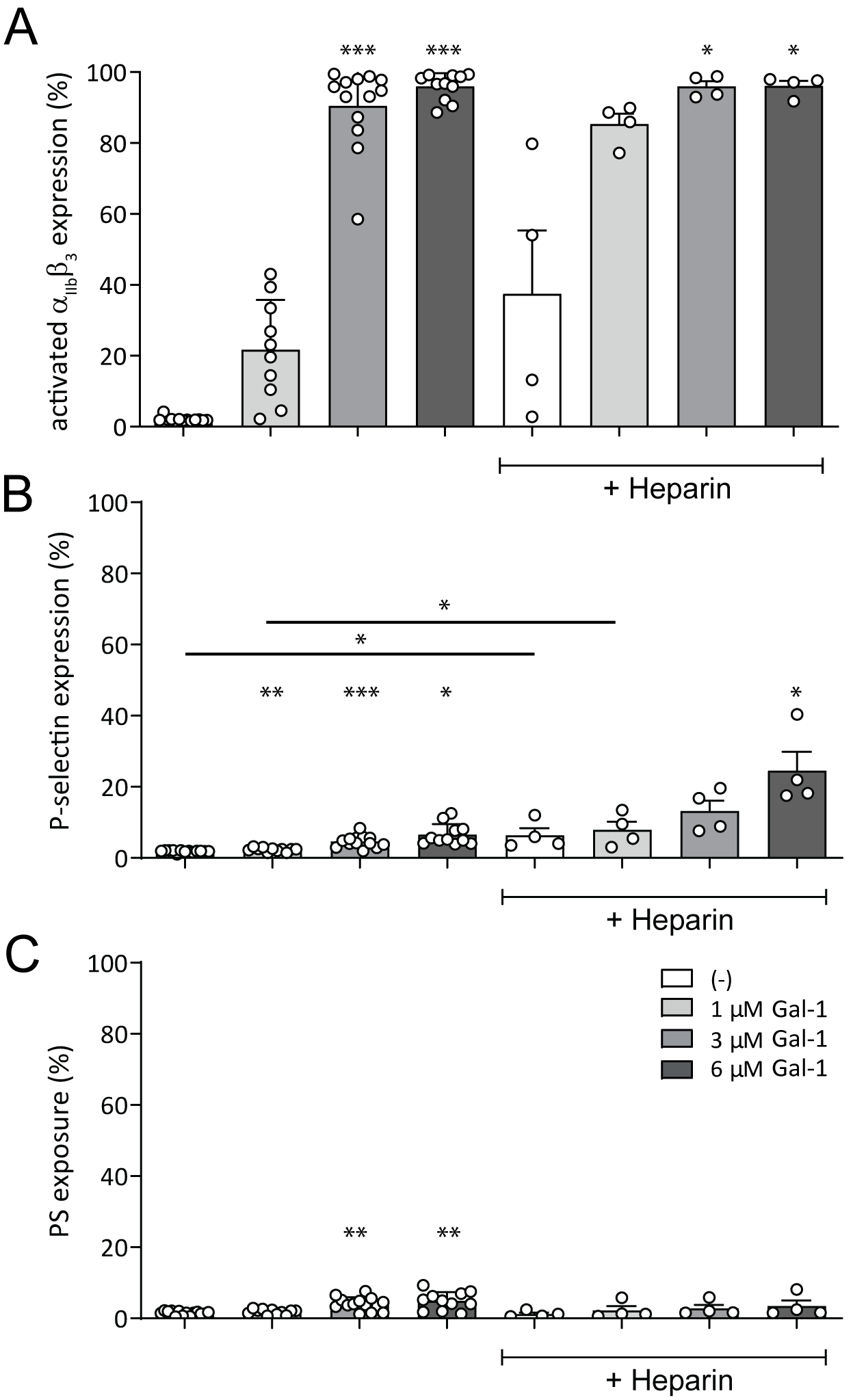


**S1 Fig: Galectin-1-induced platelet responses in the presence of heparin**.

Bar graphs represent the percentage of platelet α_IIb_β_3_ activation **(A)**, P-selectin expression **(B)** and PS-exposure **(C)** by gal-1 in the absence or presence of heparin (10µg/mL), determined by flow cytometry. Mean±SD (n=4-12). *p<0.05, **p<0.01, ***p<0.001 as compared to control (no gal-1, Kruskal Wallis/Dunn's test).
