# Supplementary material for: Galectin-1 and platelet factor 4 (CXCL4) induce complementary platelet responses in vitro
Source: PLoS One. 2021 Jan 7;16(1):e0244736. doi: 10.1371/journal.pone.0244736 (PMC7790394; doi:10.1371/journal.pone.0244736)
Supplement: S2 Fig — Platelet aggregation was induced in PRP by increasing concentrations of gal-1 or 5 μg/mL collagen. Bars represent mean ± SD (n = 3). (DOCX) [file pone.0244736.s002.docx]

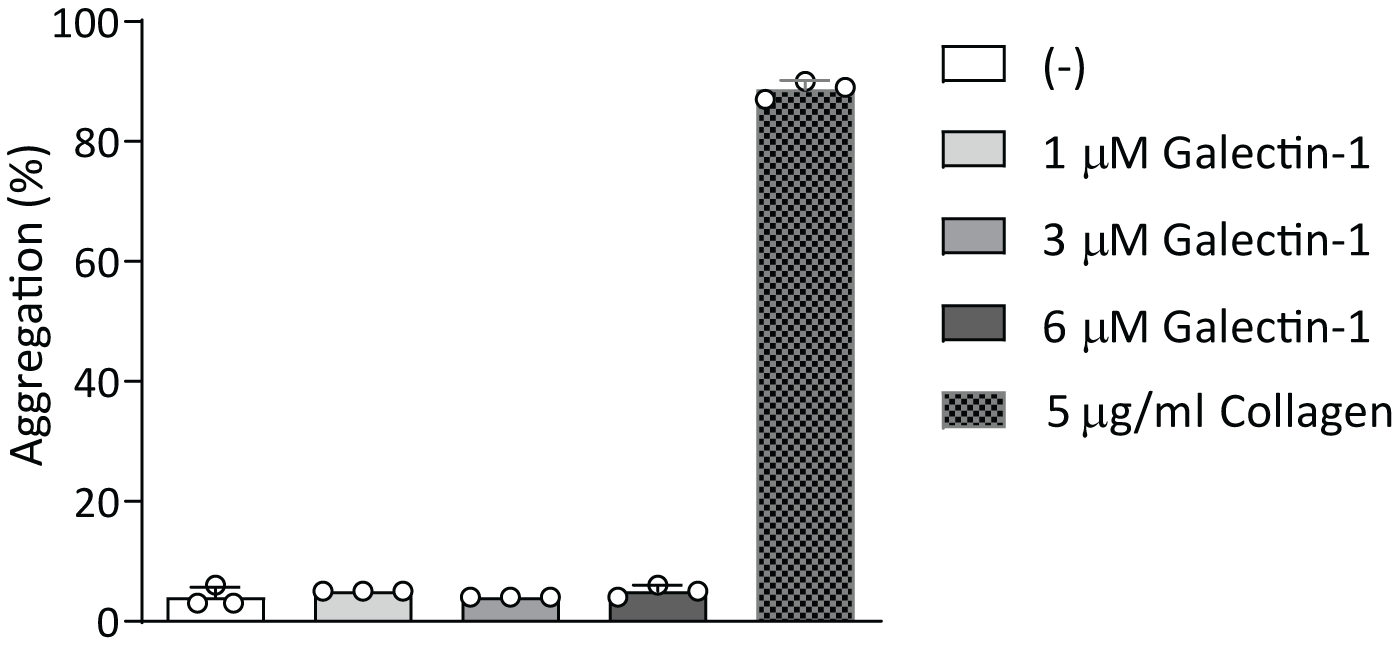


**S2 Fig: Platelet aggregation in platelet-rich plasma.** Platelet aggregation was induced in PRP by increasing concentrations of gal-1 or 5 µg/mL collagen. Bars represent mean±SD (n=3).
