# Supplementary material for: Galectin-1 and platelet factor 4 (CXCL4) induce complementary platelet responses in vitro
Source: PLoS One. 2021 Jan 7;16(1):e0244736. doi: 10.1371/journal.pone.0244736 (PMC7790394; doi:10.1371/journal.pone.0244736)
Supplement: S3 Fig — SPR analysis of CXCL4 binding on immobilized unfractionated heparin in the absence (A) or presence (B) of 500 nM gal-1. C: Sensorgram of the experimental course of KKO binding to CXCL4/heparin. CXCL4 was immobilized (i), obtaining a stable baseline (x), then KKO was perfused (ii) followed by a dissociation phase (iii). The arrows denote start and end of KKO perfusion and start of the wash phase with perfused heparin. Inset: Binding of KKO to heparin alone. D: Binding response (in resonance units, RU) of increasing concentrations of KKO (0-125 nM) in the absence (black dots) or presence (red squares) of gal-1 (500 nM). Representative sensorgrams of KKO binding to CXCL4/heparin in the absence (E) or presence (F) of gal-1. (DOCX) [file pone.0244736.s003.docx]

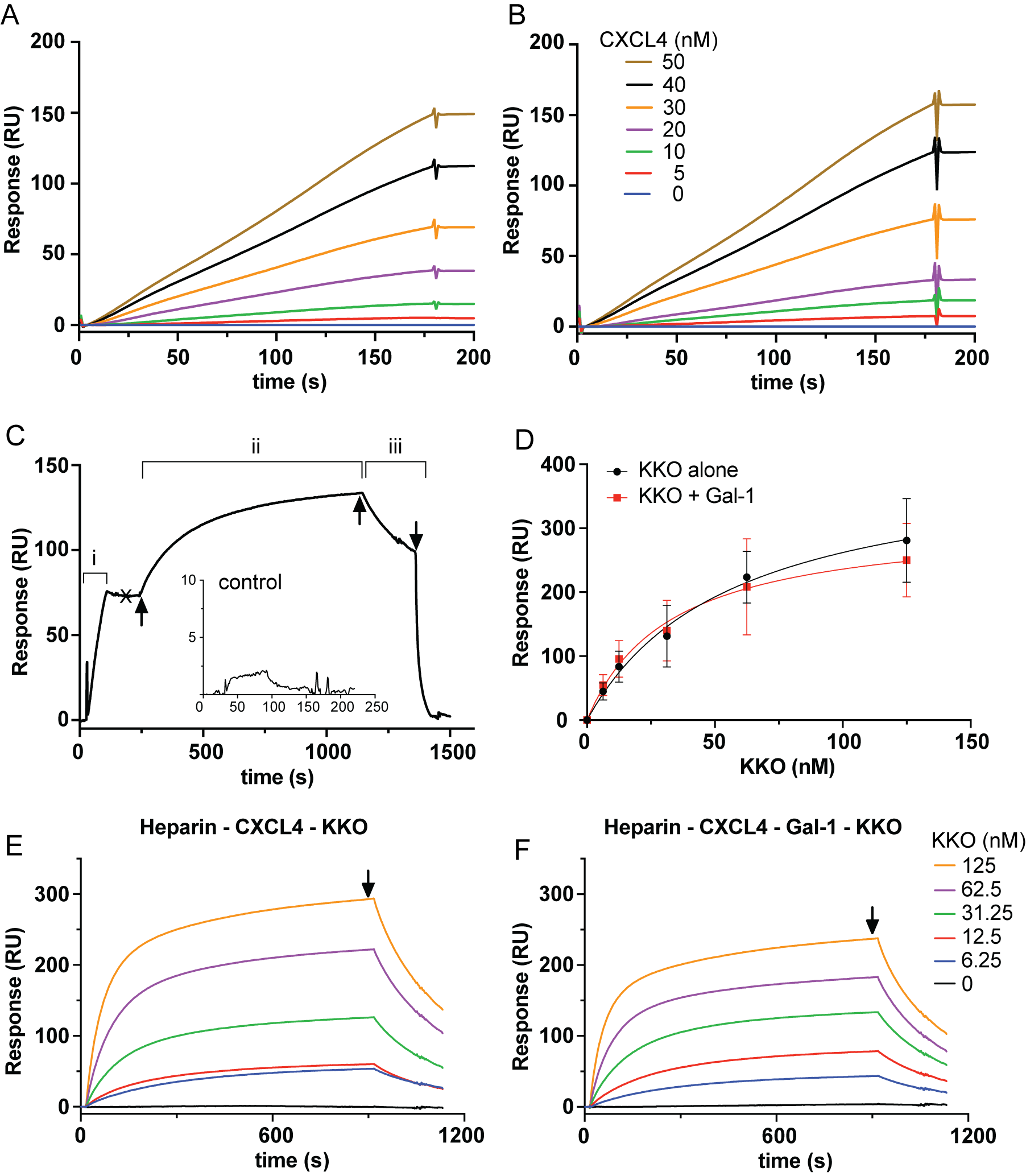


**S3 Fig: Surface plasmon resonance of CXCL4 and antibody KKO on heparin**. SPR analysis of CXCL4 binding on immobilized unfractionated heparin in the absence (**A**) or presence (**B**) of 500 nM gal-1. **C:** Sensorgram of the experimental course of KKO binding to CXCL4/heparin. CXCL4 was immobilized (i), obtaining a stable baseline (x), then KKO was perfused (ii) followed by a dissociation phase (iii). The arrows denote start and end of KKO perfusion and start of the wash phase with perfused heparin. Inset: Binding of KKO to heparin alone. **D**: Binding response (in resonance units, RU) of increasing concentrations of KKO (0-125 nM) in the absence (black dots) or presence (red squares) of gal-1 (500 nM). Representative sensorgrams of KKO binding to CXCL4/heparin in the absence (**E**) or presence (**F**) of gal-1.
